# Long-term testicular changes caused by the temporary suspension of puberty initiated at different stages of development

**DOI:** 10.3389/fcell.2026.1936689

**Published:** 2026-08-31

**Authors:** Christian Mancilla-Martínez, Itzel Jatziri Contreras-García, Julio César Rojas-Castañeda, Daniel Adrian Landero-Huerta, Rafael Reynoso-Robles, Juan Osvaldo Cuevas-Alpuche, Rosa María Vigueras-Villaseñor

**Affiliations:** 1 Maestría en Biología de la Reproducción Animal, Universidad Autónoma Metropolitana, Unidad Iztapalapa, Ciudad de México, Mexico; 2 Laboratorio de Biología de la Reproducción, Instituto Nacional de Pediatría, Ciudad de México, Mexico; 3 Laboratorio de Morfología Celular y Tisular, Instituto Nacional de Pediatría, Ciudad de México, Mexico; 4 Servicio de Urología, Instituto Nacional de Pediatría, Ciudad de México, Mexico

**Keywords:** leuprolide acetate, Leydig cell, puberty, seminiferous epithelium, Sertoli cell, testis, GnRH analog

## Abstract

**Background:**

During puberty, secondary sexual characteristics develop alongside processes of testicular cell differentiation and maturation. Puberty suppression has been introduced to prevent the development of these physical changes in some individuals who request it, as is the case with gender dysphoria. However, not everyone continues with the treatment. Therefore, the objective of this study was to determine the testicular changes caused by the temporary suppression of puberty, initiated at different stages of development, in an experimental model.

**Material and Methods:**

A total of 56 male rats were used, distributed equally into four study groups. A Control group, which received saline, and three experimental groups that received the GnRH analog (GnRHa), leuprolide acetate at 25 μg/kg/day. In the first of these groups, administration of the analog began during early puberty (LeuEP) on day postpartum (dpp) 25, equivalent to Tanner stages II-III in humans; in the second group, administration began during advanced puberty (LeuAP) at 35 dpp, equivalent to Tanner stages IV-V; and in the postpubertal group (LeuPP), administration began at 60 dpp. Half of the number of animals in each group were euthanized at 90 dpp, while the remaining animals had the GnRHa removed to allow recovery and were euthanized at 190 dpp. Testicular tissue was collected to assess histological and ultrastructural changes, as well as proteins that regulate the functional activity of Sertoli and Leydig cells.

**Results:**

The LeuEP and LeuAP groups exhibited seminiferous tubules atrophy with peritubular cell hyperplasia, resulting in thickened tubular walls. They also showed Leydig cell hyperplasia with low immunoreactivity to the HSD17B3 enzyme, reduced immunoreactivity to androgen receptor proteins, and vimentin disorganization. These same groups showed partial recovery at 190 dpp, without reaching the values of the Control group. This may have been due to the persistence of thick tubular walls, possibly resulting from the dysfunction of Leydig cells and peritubular cells, which impeded the paracrine action of molecules necessary to maintain spermatogenesis. Furthermore, this was compounded by Sertoli cell dysfunction.

**Conclusion:**

Therefore, the temporary arrest of puberty initiated during puberty resulted in permanent testicular alterations, unlike in animals where GnRHa administration began postpubertally.

## 1 Introduction

Gender dysphoria (GD) is defined as a condition in which gender incongruence (a condition in which a person’s gender identity does not align with the gender at birth) causes negative emotional distress due to perceived inconsistencies between biological sex and gender identity, and is often accompanied by significant distress. The prevalence of GD in infants and adolescents has been reported to range from 0.6% to 1.7%, and it has been steadily increasing (Wood et al., 2013; Zucker, 2017; de Graaf et al., 2018). The symptoms of GD manifest at different stages of development but become more severe during puberty, when secondary sexual characteristics develop (Atkinson and Russell, 2015). For this reason, treatments to arrest puberty have been introduced with the aim of preventing the development of secondary sexual phenotypic characteristics (Cohen-Kettenis et al., 2008).

For pubertal individuals with GD, this treatment offers the opportunity to have more time to make decisions. The use of GnRH analogs (GnRHa), such as leuprolide acetate, to suppress gonadotropins and thereby halt the progression of puberty has been documented (Pasquino et al., 2008). The Endocrine Society’s Clinical Practice Guideline (Hembree et al., 2017) recommends initiating treatment once a multidisciplinary team of medical and mental health professionals has confirmed the persistence of GD and sufficient mental capacity to provide informed consent, which most adolescents possess by age 16 (approximately Tanner stages III to IV), to allow children to experience the changes of their own pubertal development (Hembree et al., 2017). However, the optimal stage or minimum age for initiating treatment remains a subject of debate (Hembree et al., 2017). There are reports of treatment initiation as early as age 9 (Ludvigsson et al., 2023). However, not all pubertal individuals with GD decide to continue with puberty suppression and some discontinue it (Giovanardi, 2017; Brik et al., 2020). The consequences of using GnRHa to arrest puberty have been considered minor, as the effects are reportedly reversible and puberty resumes upon discontinuation of the medication (Guss and Gordon, 2022). However, these claims have recently come under scrutiny (Landén, 2024; Tornese et al., 2025). Therefore, prior to initiating treatment, individuals must be informed of potential consequences for adult fertility, given the current lack of robust evidence on this matter.

The reactivation of the hypothalamic-pituitary-testicular (HPT) axis triggers various molecular and hormonal pathways to initiate spermatogenesis. For this to occur, it is necessary for Sertoli, Leydig, and myoid peritubular cells to differentiate and mature during this phase of development.

Sertoli cells mature asynchronously (Guo et al., 2020); during this process, they increase the expression of their androgen receptors (AR) (Al-Attar et al., 1997; Berensztein et al., 2006; Rey, 2021) and reduce the production of anti-Müllerian hormone (AMH) (Edelsztein et al., 2018; Rey et al., 2009; Rey, 2021) as well as their proliferation (Meroni et al., 2019; Grinspon and Urrutia, 2020). The components of tight junctions between Sertoli cells begin to assemble between 15 and 20 days postpartum (dpp), and the cell becomes functional at 25 dpp in rats (Mok et al., 2011). Claudin-11 and connexin-43U are the main components of tight junctions and gap junctions, respectively. Their expression increases with the progression of Tanner stages in humans and is associated with a decrease in AMH concentrations (de Michele et al., 2018). Finally, the Sertoli cell assumes its final scaffold form to support the germ cells, the tubular lumen is formed, and the molecules essential for successful spermatogenesis are produced (Rey, 2014; Rey, 2021; Guo et al., 2020).

In turn, peritubular or perivascular Leydig stem cells differentiate into Leydig progenitor cells, at approximately 10 dpp in rats and persist until 28 to 36 dpp, which is when the first immature adult Leydig cells are observed. These cells change shape from elongated (stem and progenitor) to rounded interstitial (immature and mature adult) and gradually lose their proliferative capacity, while their lipid droplets become smaller. Leydig progenitor cells begin to express certain steroidogenic enzymes, such as Cyp11a1, Hhsd3b, Cyp17a1, and truncated Lhcgr (Ge and Hardy, 1998), and their proliferation is reduced in the presence of LH and androgens produced by more mature adult Leydig cells (O’Shaughnessy et al., 2019). Around dpp 35, half of the adult Leydig cell population is immature and the other half is mature (Hardy et al., 1989). By approximately day 56, mature adult Leydig cells are already present and predominant (Li et al., 2022; Wang et al., 2026). These developmental processes of Leydig cells during puberty also occur in humans (Ye et al., 2017).

In addition, androgens and FSH induce the maturation of myoid peritubular cells through the expression of actin, as described in primates (Schlatt et al., 1993; Mayer et al., 2018).

Therefore, the objective of this study was to determine the testicular changes caused by the temporary suspension of puberty, initiated at different stages of development (corresponding to Tanner stages II-V), in an experimental model. This could contribute to our understanding of the most suitable time to temporarily arrest puberty while minimizing long-term adverse effects as much as possible.

## 2 Materials and methods

### 2.1 Animals, experimental design, and sample collection

A total of 56 male rats Wistar were used. The animals were housed under a 12-h light −12-h dark cycle, with air conditioning maintained at an ambient temperature of 21 °C ± 1 °C. The rats had free access to food (Laboratory Rodent Diet 5,010, PMI Nutrition) and water. All animals received environmental enrichment and were handled regularly by the researcher. The animals were treated in accordance with the official Mexican standard (SAGARPA NOM-062-ZOO-1999) “Technical Specifications for the Production, Care, and Use of Laboratory Animals,” as well as with the internal regulations on ethical principles and the treatment of animals established by the Institutional Committee for the Care and Use of Laboratory Animals at the National Institute of Pediatrics, Mexico City, Mexico, registration number INP 2024/005.

The animals were randomly assigned in equal numbers to 1 Control group and three experimental groups. Animals in group 1 (Control group) received a daily administration of 0.9% physiological saline solution (SS, 1 mL/kg s. c.); while animals in groups 2 through 4 (experimental) received a daily dose of leuprolide acetate dissolved in SS (25 μg/kg/day s. c.; Santa Cruz Biotechnology sc-394418) (Lehrer et al., 1992; Guarraci et al., 2021). All administrations were carried out daily at approximately 10 a.m. Group 2 (LeuEP) began treatment at 25 dpp (early puberty), equivalent to Tanner stages II-III in humans; group 3 (LeuAP) at 35 dpp (advanced puberty), equivalent to Tanner stages IV-V; and group 4 (LeuPP) at 60 dpp (postpubertal stage) (Guo et al., 2020; Rey, 2021). SS and leuprolide acetate were administered daily and continued until 90 dpp, an age equivalent to 18 years in humans in terms of testicular development. Half of the number of animals in each group were euthanized at 90 dpp, while the remaining animals had the drug withdrawn; they were allowed to recover over two spermatogenic cycles and were finally euthanized at 190 dpp. Euthanasia was performed using an overdose of pentobarbital (120-150 mg/kg i. p.) diluted with normal saline in a 1:1 ratio to reduce irritation caused by drug administration. Subsequently, the testes of each of the animals were extracted and weighed to obtain the relationship with respect to body weight, gonadosomatic index.

### 2.2 Morphological analysis

The right testis was fixed in modified Karnovsky’s solution (Karnovsky, 1965) for morphological studies, and the left testis was fixed in 4% paraformaldehyde for immunohistochemical studies.

After 24 h of fixation in modified Karnovsky’s solution, the testes were postfixed with 1% osmium tetroxide (Merck, Darmstadt, Germany). Semi-thin sections, 1 μm thick, were obtained with an ultramicrotome (Leica Ultracut-UCT, Vienna, Austria), stained with 0.5% toluidine blue (Sigma-Aldrich, Mexico), and finally mounted on slides.

The histological analysis was performed by a single observer using an image analysis system (Image-Pro Plus 6.0) and an optical microscope (BX 51, Olympus). For each testis, at least 15 cross-sections of seminiferous tubes were examined (discarding tubes cut with oblique or longitudinal orientations) to determine the following: a) area of the seminiferous tubes, b) maturation of the seminiferous epithelium or Johnsen index (Johnsen, 1970), c) histological alterations (Vigueras-Villaseñor et al., 2009), d) thickness of the seminiferous tubes wall, composed of cellular layers and the basement membrane surrounding each seminiferous tube, e) number of mast cells per interstitium and f) number of Leydig cells per interstitium. The numbers of mast cells and Leydig cells were determined within the interstitial space, specifically at the centroid of the triangle formed by three adjacent seminiferous tubules, across an area of 16,000 μm^2^. This was performed in four different interstitial spaces within the same animal. Based on these data and taking into account the Control group mean ± 1SD, the following variables were defined for statistical analysis: a) Percentage of atrophic tubes (%Atrophy), those with an area value <72,400 μm^2^/tube at 90 dpp and <74,000 μm^2^/tube at 190 dpp; b) Percentage of tubes with arrested spermatogenesis (%Arrested Spermatogenesis), those with a seminiferous epithelium maturation grade <8/tube; c) Percentage of tubes with histological abnormalities (%Damage), those with a value of >3/tube; and d) Percentage of tubes with thick walls (%Tubes with thick walls), those with a value >2.17 μm/tube for 90 dpp and 2.36 μm/tube for 190 dpp.

To confirm the histological findings, ultrafine sections (70 nm thick) were made, mounted on copper grids and contrasted with uranyl acetate and lead citrate. These were later observed in an electron microscope (JEM-1011, JEOL, Osaka, Japan).

#### 2.2.1 Determination of cells in apoptosis

Testicular samples fixed in 4% paraformaldehyde were kept in this solution for 18 h and processed for paraffin embedding. Sections of 5 μm thickness were cut and mounted on slides with poly-L- lysine (Sigma-Aldrich, Mexico). The slides were deparaffinized and hydrated in a series of alcohols and incubated in a 0.1% Triton permeabilizing solution. They were washed with PBS and subsequently incubated in TUNEL solution (Roche Diagnostic Corporation, USA). Finally, they were washed with PBS and mounted with a fluorescence mounting medium (Fluoroshield Mounting Medium with DAPI, ABCAM) for observation under a fluorescence microscope (Olympus BX51). The number of apoptotic cells was assessed per cross-section in at least 15 seminiferous tubes per animal (Li et al., 2023). The percentage of seminiferous tubes undergoing apoptosis (%Apoptosis) was determined by considering those with a value of >2 apoptotic cells/tube at 90 dpp and >1 apoptotic cell/tube at 190 dpp, as indicated by the mean + 1SD value of the Control group.

#### 2.2.2 Staining technique for collagen fibers

The paraffin-embedded sections were cut to a thickness of 5 μm and stained using the Masson’s staining technique. Briefly, they were post-fixed in a Bouin’s solution with acetic acid at room temperature overnight. The following day, they were washed with distilled water and stained with Weigert’s ferric hematoxylin, followed by scarlet fuchsin, then a phosphomolybdic-phosphotungstic acid solution, and finally an aniline blue solution. Between each step, the sections were washed with distilled water. The tissue sections were mounted on glass slides and examined under a microscope. The collagen fibers stained blue.

### 2.3 Immunohistochemical analysis for AR, vimentin, and HSD17B3

From the paraffin-embedded samples, 5 μm-thick sections were prepared, deparaffinized, hydrated, and incubated with sodium citrate (0.01 M, pH 7.6) for 5 min in a microwave (800 W). Subsequently, they were incubated with bovine serum before being treated with the rabbit polyclonal primary antibody against the androgen receptor, a protein that regulates the functional activity of Sertoli cells (sc-816, Santa Cruz Biotechnology) at a 1:50 dilution. The mouse monoclonal primary antibody against the vimentin, a structural protein that also allows for the determination of Sertoli cell function (sc-6260, Santa Cruz Biotechnology), was used at a 1:200 dilution. The rabbit polyclonal primary antibody against HSD17B3 enzyme characteristic of the final phase of steroidogenesis, at a 1:200 dilution. (OAGA04684, Aviva System Biology). Biotinylated secondary antibodies diluted in bovine serum were used depending on the species of the primary antibody (Goat anti-rabbit BA-1000, Vector Labs, and Horse anti-mouse IgG Biotinylated BA-2000, Vector Labs, Burlington, ON, Canada, respectively) at a 1:200 dilution for 2 h at room temperature. The tissues were incubated with the avidin-biotin conjugate system (Vectastain ABC kit, PK-6100, Vector Labs) for 1 h at room temperature and were evidenced with diaminobenzidine (DAB Chromogen Kit DS854H, Biocare, Concord, CA, United States), counterstained with Gill’s hematoxylin, and mounted with Entellan (Merck). The androgen receptor immunoreactive cell number (ARICN) per cross-section was determined in at least 15 seminiferous tubes, in stages III to VII of the seminiferous epithelium cycle. The percentage of seminiferous tubules with ARICN reduction (%ARICN reduction) was determined with a value <41 immunoreactive cells/tube for 90 dpp and <46 immunoreactive cells/tube for 190 dpp which correspond to the average values of the Control group -1SD.

### 2.4 Statistical analysis

The results were analyzed using the Shapiro-Wilk test, followed by ANOVA and a Tukey *post hoc* test for multiple comparisons at a significance level of p < 0.05. A Student’s t-test was used to compare the ages at 90 vs. 190 dpp.

## 3 Results

### 3.1 Analysis at 90 dpp

#### 3.1.1 Gonadosomatic index and morphometric parameters

A significant reduction in the gonadosomatic index was observed in the LeuEP and LeuAP groups (p < 0.05) but not in the LeuPP group (p > 0.05) when compared to the Control group (Table 1).

**TABLE 1:** Percentage of damaged tubes for each parameter evaluated at 90 dpp.

Groups	Gonadosomatic index	Atrophy (%)	Arrested spermatogenesis (%)	Damage (%)	Tubes with thick walls (%)	Apoptosis (%)	ARICN reduction (%)
Control	0.46 ± 0.04	30.86 ± 6.14	0.40 ± 1.37	0	18.24 ± 6.03	09.34 ± 7.58	20.67 ± 7.22
LeuEP	0.21 ± 0.06^ad^	98.01 ± 3.21^ad^	89.25 ± 10.87^acd^	93.82 ± 8.34^acd^	89.96 ± 6.08^ad^	97.43 ± 5.75^ad^	100.00^a^
LeuAP	0.30 ± 0.09^ad^	90.04 ± 4.01^ad^	61.48 ± 19.83^abd^	65.85 ± 7.94^abd^	83.67 ± 2.76^a^	75.89 ± 11.96^a^	100.00^a^
LeuPP	0.37 ± 0.07^b^	70.93 ± 15.00^abc^	21.43 ± 30.25^bc^	29.69 ± 19.74^abc^	74.37 ± 10.86^ab^	56.86 ± 24.32^ab^	100.00^a^

Results are expressed as mean ± SE. ^a^ Vs. Control (p < 0.05), ^b^ Vs. LeuEP (p < 0.05), ^c^ Vs. LeuAP (p < 0.05), ^d^ Vs. LeuPP (p < 0.05). LeuEP, leuprolide early puberty, LeuAP = leuprolide advanced puberty, LeuPP, leuprolide postpubertal; ARICN , androgen receptor immunoreactive cell number.

Upon quantifying the morphometric parameters and comparing them with the Control group, all experimental groups showed significant changes in %Atrophy and %Damage (p < 0.05). For the %Arrested Spermatogenesis variable, the LeuEP and LeuAP groups were higher than the Control group (p < 0.05). Regarding %Tubes with thick walls, this value was higher in all experimental groups compared to the Control (p < 0.05). The most affected group was LeuEP, and the least affected was LeuPP (Table 1, p < 0.05).

#### 3.1.2 Morphological parameters

**FIGURE 1:**
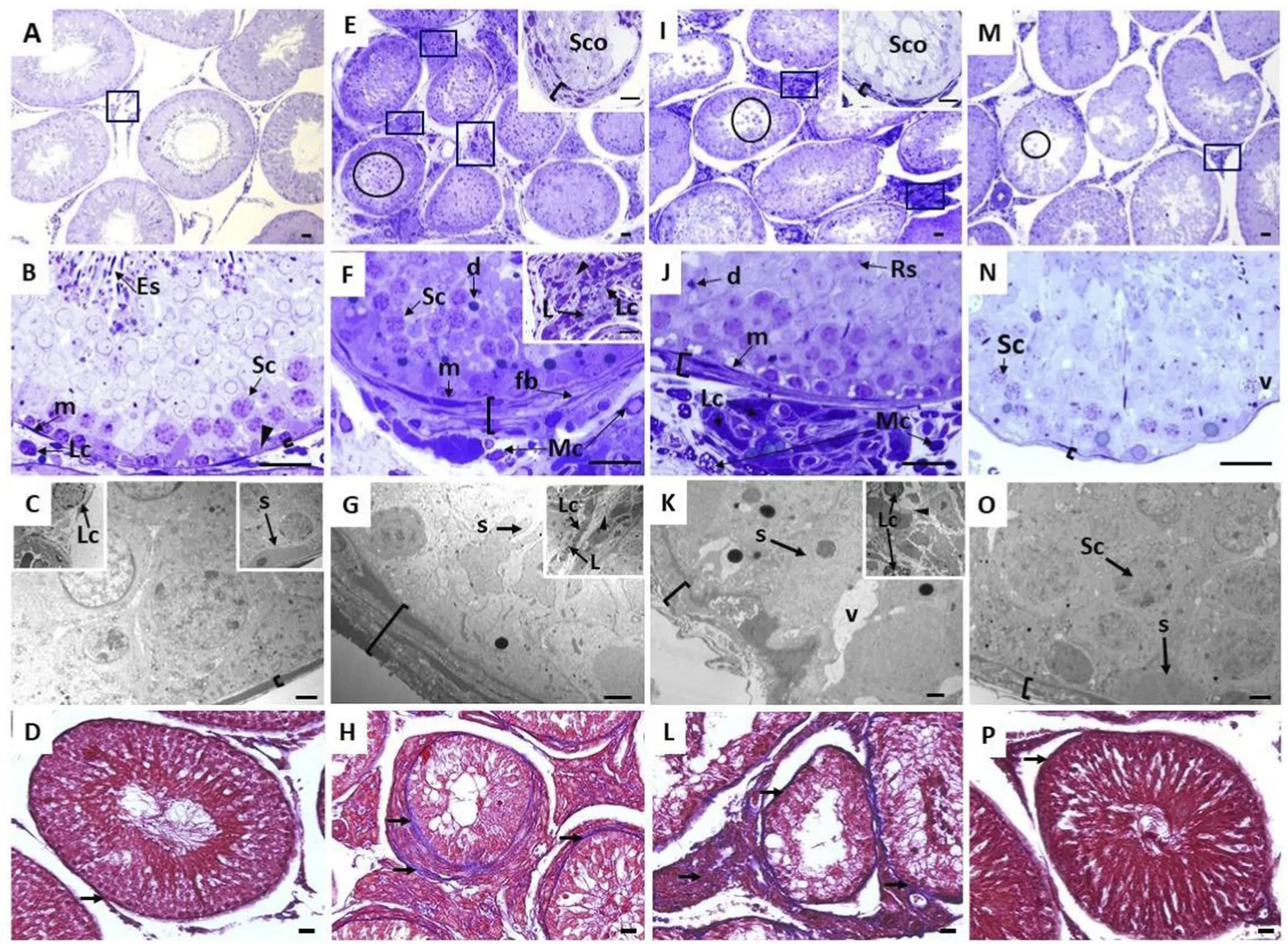
Seminiferous tubes at 90 dpp under different experimental conditions. Control group: **(A)** healthy tubes with a lumen and loosely distributed Leydig cells (inset); **(B)** at higher magnification, complete cytoarchitecture and spermatogenesis are observed with a thin tubular wall (black bracket), composed mainly of myoid peritubular cells (arrow); there are few mast cells in the interstitium; **(C)** the nuclear membrane of the Sertoli cell is visible without indentations, and Leydig cells are seen without lipid droplets (see also insets); **(D)** collagen fibers are scarce (arrow). LeuEP group: **(E)** small, atrophied seminiferous tubes are observed, with an absent lumen, cellular shedding (circle), and hyperplasia of Leydig cells (box), and some tubes containing only Sertoli cells (inset); **(F)**, at higher magnification, arrested spermatogenesis is observed, along with degenerating cells; the tubule walls appear thickened (bracket), with two morphologically distinct types of Leydig cells, one with fine lipid droplets (arrowhead) and the other with large lipid droplets (arrow) (inset), and a large number of mast cells; **(G)**, Sertoli cell nuclei with deep indentations and progenitor Leydig cells in the tubular wall with lipid droplets (arrow) are visible (inset); **(H)**, the abundant presence of collagen fibers interspersed in the tubular wall is evident (arrow). LeuAP group **(I,J,K,L)**: the same changes as in the LeuEP group are observed, but to a lesser extent. LeuPP group **(M,N,O,P)**: the changes described for the LeuAP group were observed to a lesser degree. (Sc) Spermatocytes, (Rs) round spermatids, (Es) elongated spermatids, (s) Sertoli cell nuclei, (m) myoid peritubular cells, (Lc) Leydig cells, (d) degenerating cells, (v) vacuoles, **(L)** lipid droplets. Toluidine blue **(A,B,E,F,I,J,M,N)**, scale bar: 20 μm. Electron microscopy **(C,G,K,O)**, scale bar: 2 μm. Masson’s stain **(D,H,L,P)**. Scale bar: 20 μm.

**FIGURE 2:**
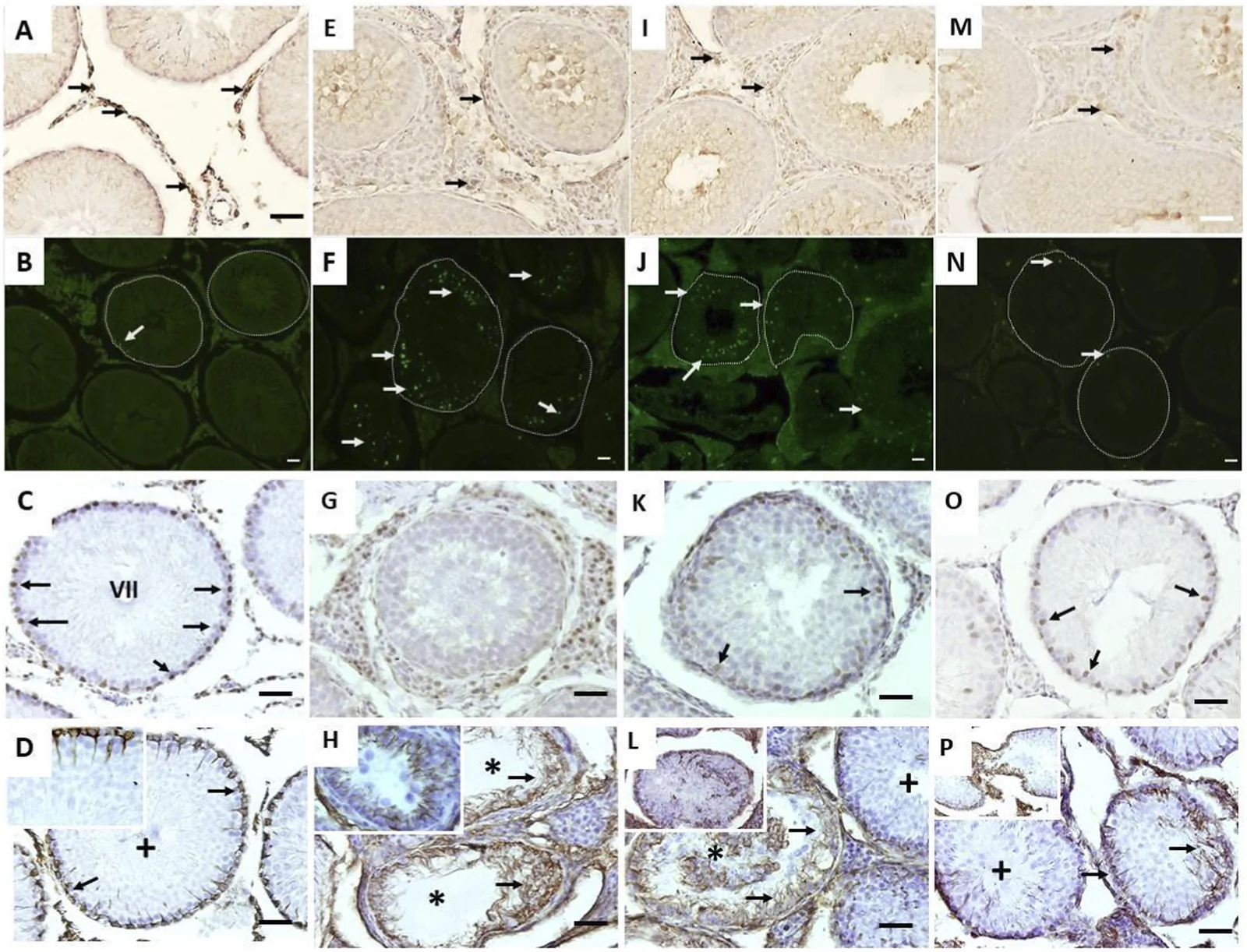
Seminiferous tubes at 90 dpp. Immunohistochemistry for the HSD17B3 enzyme: in the Control group **(A)**, Leydig cells immunoreactive to HSD17B3 (arrow) are observed; in the LeuEP, LeuAP, and LeuPP groups at 90 dpp **(E,I,M)** respectively, immunoreactivity for the enzyme is scarce (arrow). TUNEL technique to identify cells undergoing apoptosis: in the Control group **(B)**, no cells undergoing apoptosis are observed; in the LeuEP and LeuAP groups **(F,J)**, a large number of cells undergoing apoptosis are observed (arrow); whereas in the LeuAP group **(N)**, these are scarce (arrow). Immunohistochemistry for AR: in the Control group **(C)**, the presence of AR in the seminiferous tubule is clear; in the LeuEP and LeuAP groups **(G,K)**, cells immunoreactive to AR are scarce; the LeuPP group **(O)** showed a greater number of cells immunoreactive to AR compared to LeuEP and LeuAP. Immunohistochemistry for vimentin: in the Control group **(D)**, an immunoreaction perpendicular to the basement membrane is observed, with microfilaments extending toward the tubular lumen; the experimental groups showed disorganization of vimentin; the severity of this disorganization depended on the timing of leuprolide administration, being most severe in the LeuEP group **(H)**, followed by LeuAP **(L)**, and finally, LeuPP **(P)**. The stages of the seminiferous epithelium cycle are shown in roman numerals. Scale bar: 50 μm.

**FIGURE 3:**
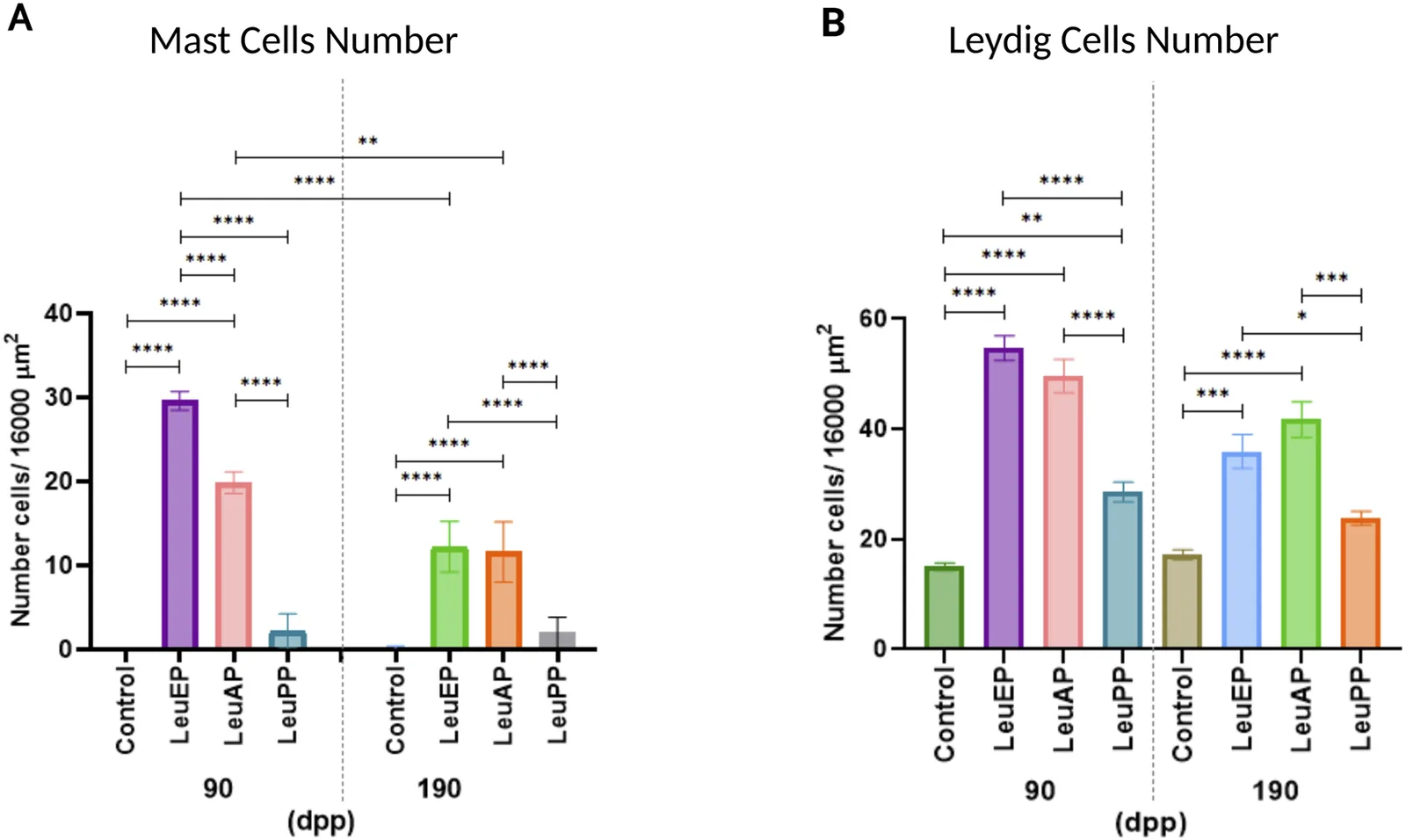
The number of mast cells **(A)** and Leydig cells **(B)** at 90 and 190 dpp under the different experimental conditions is shown. ANOVA and Tukey’s test were used for multiple group comparisons by age, and Student’s t-test was used to compare 90 vs. 190 dpp. *p < 0.05, **p < 0.01, ***p < 0.001, ****p < 0.0001.

The Control group exhibited complete spermatogenesis, with a large number of spermatozoa in the lumen of the seminiferous tubes (Figures 1A,B). Under the electron microscope, the nuclei of the Sertoli cells were observed at the periphery of the tube and showed slightly pronounced indentations (Figure 1C). Toward the periphery of the seminiferous tube, a basal lamina was observed associated with a single layer of myoid peritubular cells (Figure 1B) with no apparent increase in collagen fibers (Figure 1D). Leydig cells were present in the interstitial space, exhibiting their characteristic polyhedral shape and forming clusters of 5–7 cells (Figure 1A), with cytoplasmic extensions (Figure 1C) and showing intense immunoreactivity to the HSD17B3 enzyme (Figure 2A). The connective tissue was characterized by loose areolar tissue with sparsely distributed collagen fibers (Figure 1D), along with fibroblasts and a few interstitial mast cells (0.05 ± 0.11); and Leydig cells (15 ± 1.53; Figures 1B, 3A,B).

The LeuEP and LeuAP groups exhibited abnormalities such as arrested spermatogenesis, primarily in leptotene/pachytene spermatocytes (Figures 1E,F) and in round spermatids (Figures 1I,J), respectively, as well as a reduction in seminiferous tube size, accompanied by disorganization of the cytoarchitecture, vacuolization, cell detachment, pyknosis, germ cell hypoplasia, and, in some tubes, the absence of any type of germ cell, with Sertoli cells only (SCO) present (Figures 1E,F,I,J). Under the electron microscope, the nuclei of the Sertoli cells were observed to be slightly displaced toward the lumen, with deep atypical indentations and thick, folded basal membranes (Figures 1G,K). A thick wall was observed surrounding the seminiferous tubes. This wall was presumably composed of myoid peritubular cells, fibroblasts, collagen fibers, and apparently progenitor/immature Leydig cells identified by the presence of lipid droplets (Figures 1F–H, J–L). Occupying a large portion of the interstitium, there was clear evidence of hyperplasia of Leydig cells with mild immunoreactivity to the HSD17B3 enzyme (Figures 1E,F,I,J, 2E,I). This finding was more pronounced in the LeuEP group, where two morphologically distinct populations were observed. One was round to oval in shape with a well-defined outline and the presence of coarse lipid droplets, and the other was polyhedral in shape with fine lipid droplets (Figure 1F,J), which, under the electron microscope, were observed to have thin cytoplasmic projections in greater numbers than in the Control group (Figures 1G,K). The number of mast cells and Leydig cells were higher in the LeuEP group and the LeuAP group (29.60 ± 1.12; and 19.85 ± 1.30, respectively for mast cell; 54.71 ± 5.42 and 49.65 ± 6.75, respectively for Leydig cells; Figures 1F,J, 3A,B; p < 0.05) compared to the Control group. In fact, the LeuEP group had a higher number of mast cells than the LeuAP group (p < 0.05).

The LeuPP group also exhibited arrested spermatogenesis, with seminiferous tubes reduced in size and germ cell hypoplasia to a lesser degree than in the LeuEP and LeuAP groups. Seminiferous tubes showed cellular disorganization, vacuolization, and pyknosis (Figures 1M,N). The basement membranes were observed to be slightly thicker than in the Control group (Figures 1N,O), with the presence of myoid peritubular cells, fibroblasts, and sparse collagen fibers (Figures 1N,P). In this group, the hyperplasia of Leydig cell was greater than the Control group (28.60 ± 4.01, p < 0.05) and the number of mast cells (2.15 ± 2.08), showing no statistically significant difference (p > 0.05) compared to the Control group (Figures 1M,N; Figures 3A,B).

The severity of tubular and interstitial alterations depended on the stage at which GnRHa was administered, being greater in the LeuEP group and less in the LeuPP group.

#### 3.1.3 Apoptosis and proteins regulating Sertoli cell function

The %Apoptosis was higher in the experimental groups compared to the Control (Table 1, p < 0.05; Figures 2B,F,J,N). The ARICN in the Control group was 41.07 ± 1.62; all experimental groups showed an increase in the %ARICN reduction (p < 0.05), with an average ARICN per tube of 4.49 ± 2.76 for LeuEP, 5.31 ± 2.68 for LeuAP, and 23.18 ± 2.42 for LeuPP (Table 1, p < 0.05; Figures 2C,G,K,O).

Vimentin was distributed perpendicular to the basement membrane, forming branched structures in the Control group. In the LeuEP and LeuAP groups, this distribution was lost to varying degrees, with the loss being more pronounced in seminiferous tubes showing clear tubular atrophy. The LeuPP group retained the distribution observed in the Control group, although it exhibited areas where this distribution was lost (Figures 2D,H,L,P).

### 3.2 Analysis at 190 dpp

#### 3.2.1 Gonadosomatic index and morphometric parameters

Following the recovery period and upon comparing each group with its 90 dpp counterpart, we found that LeuEP increased the gonadosomatic index by 61.00% (p < 0.05). LeuAP and LeuPP reduced this parameter by 3.33% and 8.10%, respectively (p < 0.05). At 190 dpp, the only group that did not reach the gonadosomatic index values of the Control group was LeuAP (Table 2, p < 0.05).

**TABLE 2:** Percentage of damaged tubes for each parameter evaluated at 190 dpp.

Groups	Gonadosomatic index	Atrophy (%)	Arrested spermatogenesis (%)	Damage (%)	Tubes with thick walls (%)	Apoptosis (%)	ARICN reduction (%)
Control	0.35 ± 0.05	26.86 ± 8.58	3.42 ± 6.49	0.23 ± 0.78	24.63 ± 7.82	07.44 ± 5.15	16.67 ± 11.79
LeuEP	0.34 ± 0.02	86.55 ± 11.58^ad^	56.82 ± 26.89^ad^	51.36 ± 32.21^ad^	74.75 ± 5.58^ad^	33.67 ± 15.56^a^	100.00^a^
LeuAP	0.29 ± 0.05^a^	72.23 ± 13.94^ad^	54.38 ± 28.10^ad^	50.48 ± 20.16^ad^	73.73 ± 2.94^ad^	22.33 ± 7.24	100.00^a^
LeuPP	0.34 ± 0.04	50.15 ± 17.92^abc^	20.25 ± 21.16^bc^	13.55 ± 10.31^bc^	53.98 ± 8.76^abc^	20.08 ± 12.71	83.81 ± 14.95^a^

Results are expressed as mean ± SE. ^a^ Vs. Control (p < 0.05), ^b^ Vs. LeuEP (p < 0.05), ^c^ Vs. LeuAP (p < 0.05), ^d^ Vs. LeuPP (p < 0.05). LeuEP, leuprolide early puberty, LeuAP = leuprolide advanced puberty, LeuPP, Leuprolide postpubertal; ARICN , androgen receptor immunoreactive cell number.

**FIGURE 4:**
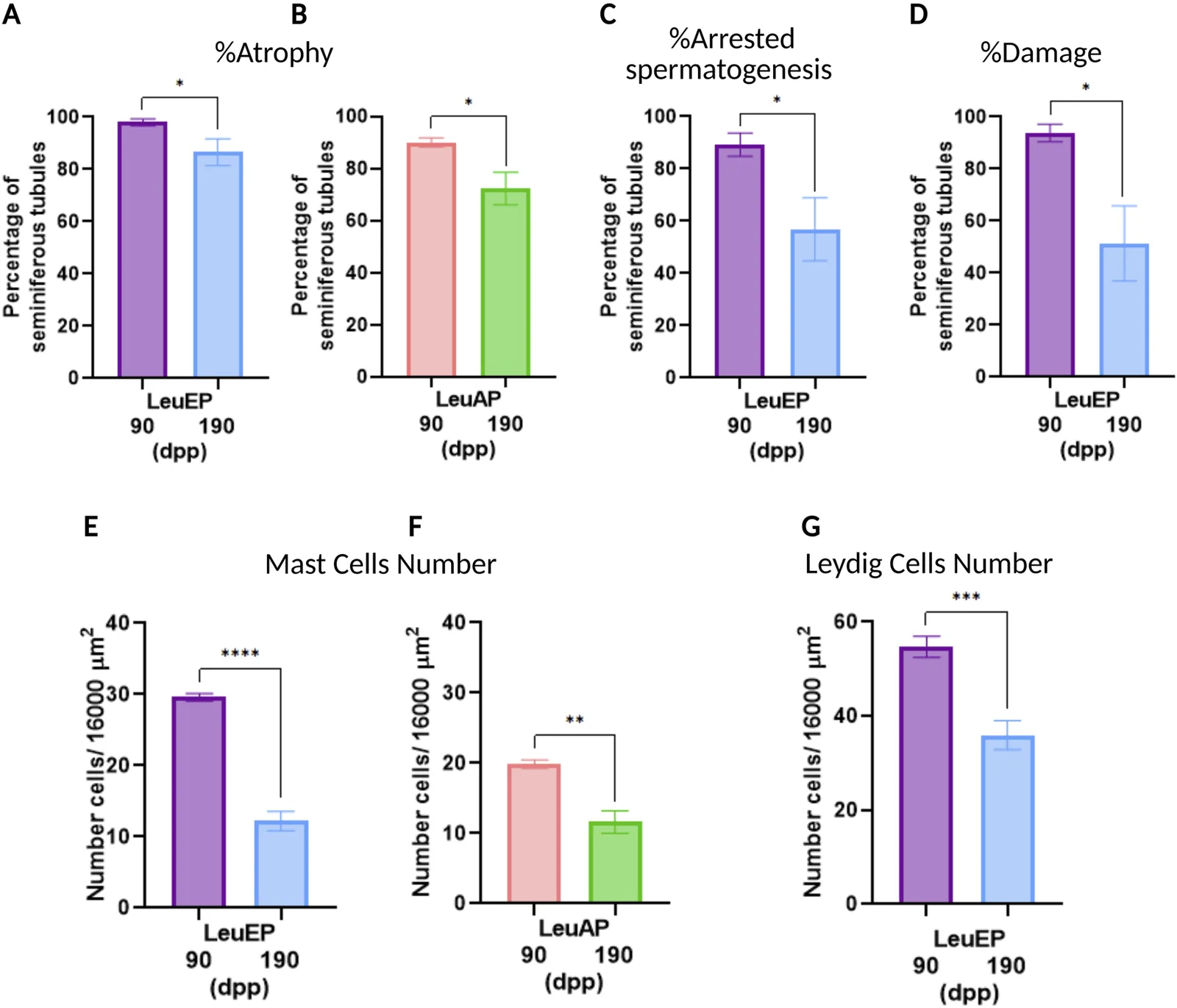
The parameters %Atrophy **(A,B)**, %Arrested Spermatogenesis **(C)**, %Damage **(D)**, and number of mast cells **(E,F)** and Leydig cells **(G)** are shown, comparing the ages of 90 dpp and 190 dpp. t-test, *p < 0.05, **p < 0.01, ***p < 0.001, ****p < 0.0001.

When comparing the analyzed parameters of each group (190 dpp) with those from 90 dpp, the LeuEP group showed a decrease in %Atrophy, %Arrested Spermatogenesis, and %Damage (Figures 4A,C,D, p < 0.05). LeuAP showed a decrease (p < 0.05) in %Atrophy (Figure 4B), and no difference in %Arrested Spermatogenesis and %Damage, compared to the values at 90 dpp. When compared to its 90 dpp predecessor, the LeuPP group showed no differences in %Atrophy, %Arrested Spermatogenesis, and %Damage.

After the recovery period, at 190 dpp in the LeuEP and LeuAP groups, the %Atrophy, %Arrested Spermatogenesis, and %Damage did not reach the values of the Control group (Table 2, p < 0.05). Thus, the LeuEP and LeuAP groups showed 51.36% and 50.48%, respectively, of damaged tubules, and of these, ≈15% exhibited SCO. As for the LeuPP group, 13.55% of its seminiferous tubes remained damaged, with no difference from the Control group, and the %Arrested Spermatogenesis was also not significantly different (p > 0.05), although 6% of the seminiferous tubes exhibited SCO. However, the %Atrophy did not reach the values observed in the Control group (Table 2, p < 0.05).

**FIGURE 5:**
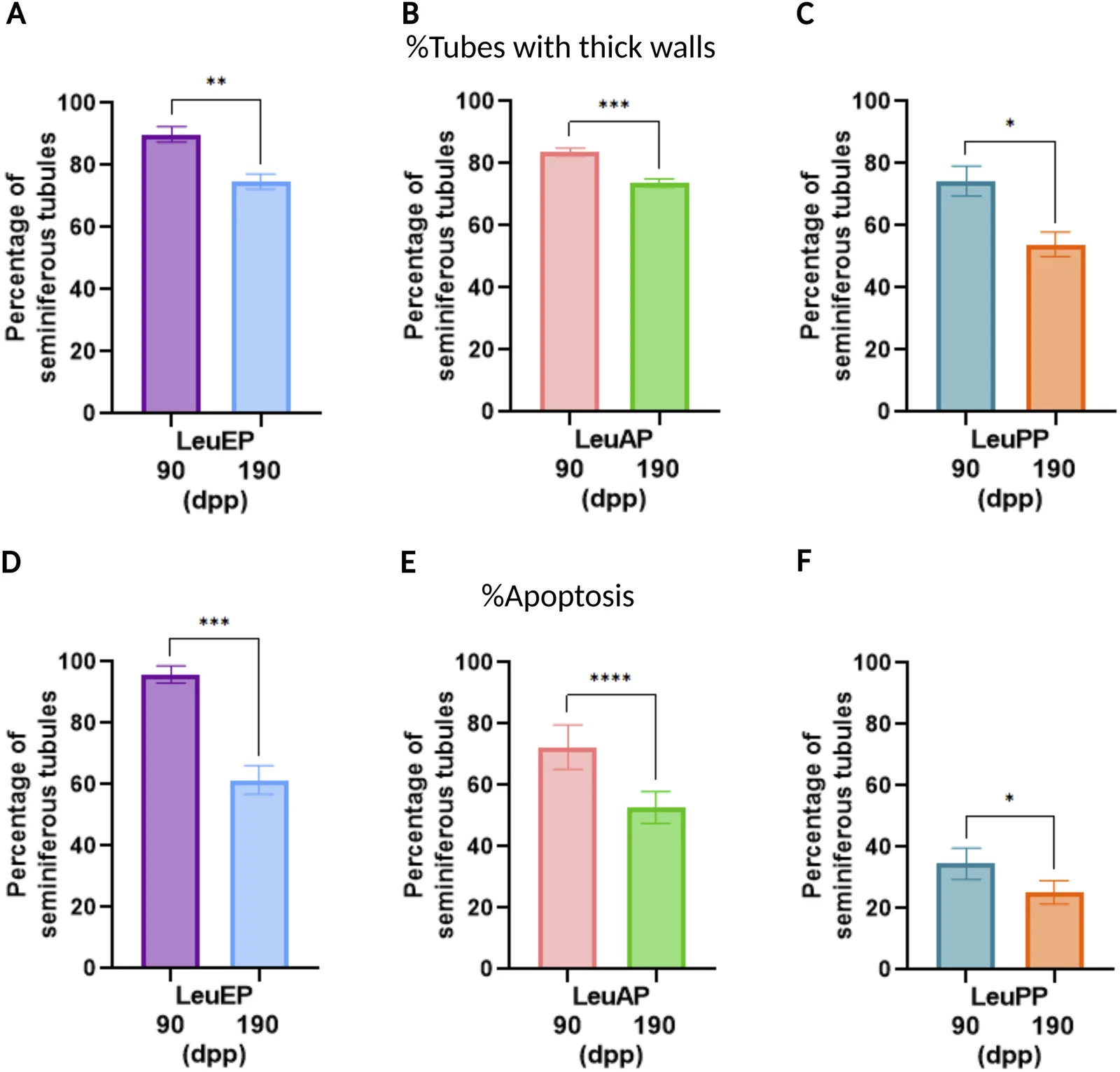
The parameters %Tubes with thick walls **(A–C)** and apoptosis **(D–F)** are shown, comparing the ages of 90 dpp and 190 dpp. t-test, *p < 0.05, **p < 0.01, ***p < 0.001, ****p < 0.0001.

The %Tubes with thick walls decreased to 190 dpp compared with 90 dpp for all experimental groups (Figures 5A–C); however, they did not reach the thickness of the Control group, particularly in the tubes that remained atrophied (Table 2, p < 0.05).

#### 3.2.2 Morphological parameters

**FIGURE 6:**
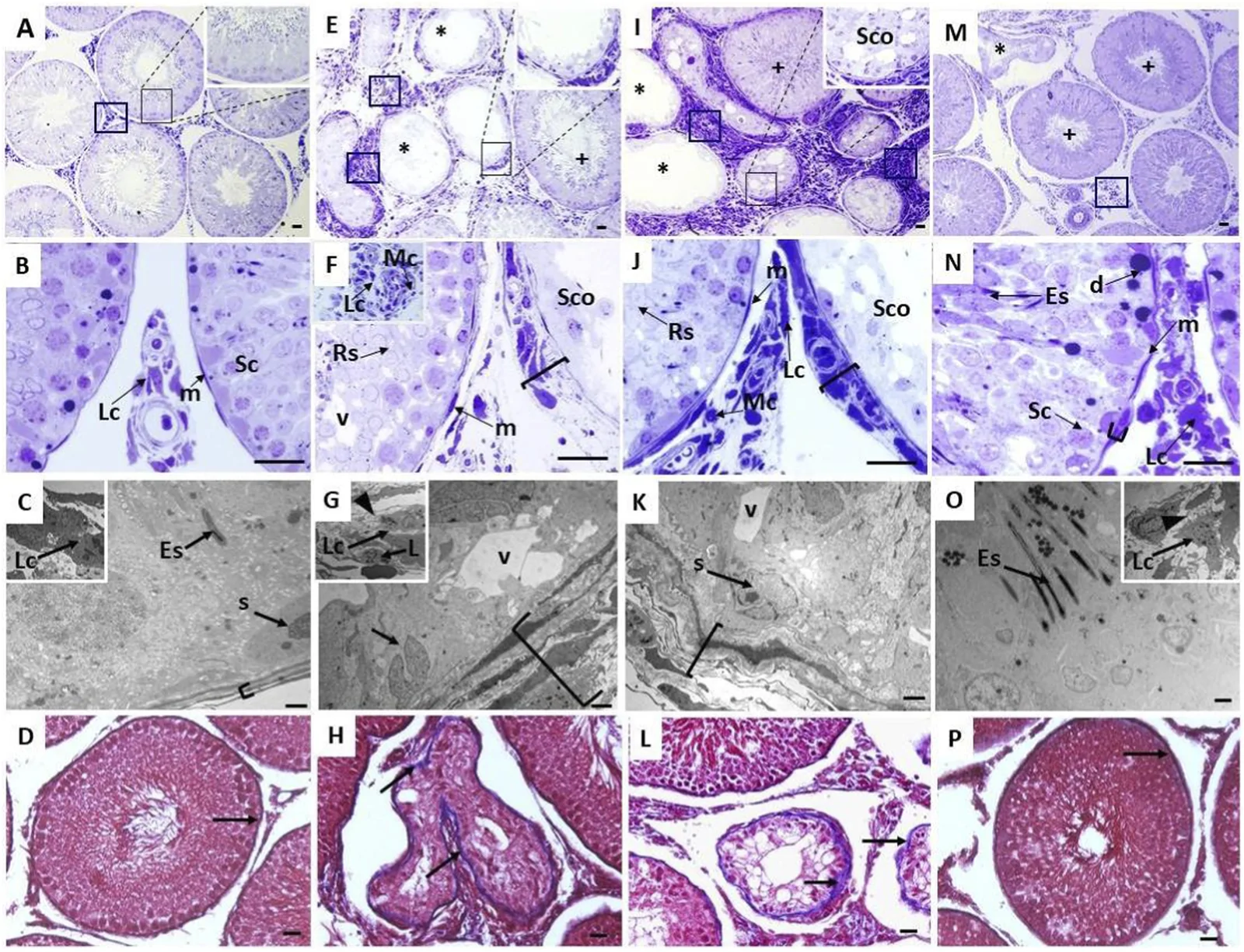
Seminiferous tubules at 190 dpp under different experimental conditions. Control group: **(A)**, normal cytoarchitecture is observed, with complete spermatogenesis and a loose distribution of Leydig cells (inset); **(B)** at higher magnification, thin tubular walls and Leydig cells in the interstitium are observed; **(C)**, Sertoli cell nuclei without indentations are observed, along with Leydig cells containing small lipid droplets and cytoplasmic extensions (inset); **(D)** with a small number of collagen fibers in the tubular wall (arrow). LeuEP group: **(E)** atrophied seminiferous tubules (*) are observed, some containing only Sertoli cells (inset), alongside tubules with complete spermatogenesis (+) and interstitial cell hyperplasia (frame); at higher magnification **(F)**, the atrophied tubes show thick tubular walls, while the tubes with spermatogenesis have thin tubular walls and atrophic Leydig cells (inset); **(G)**, Sertoli cell nuclei with deep indentations (arrow) and Leydig cells with long cytoplasmic extensions (inset G) are visible; **(H)**, a large number of collagen fibers are intercalated within the tubule wall. The LeuAP group **(I,J,K,L)**: shows the same changes as the LeuEP group, but to a lesser extent. The LeuPP group **(M,N,O,P)**: exhibited the same characteristic as the LeuAP group, but with less severity. (Sc) Spermatocytes, (Rs) round spermatids, (Es) elongated spermatids, (s) Sertoli cell nuclei, (m) myoid peritubular cells, (Lc) Leydig cells, (d) degenerating cells, (v) vacuoles, (L) lipid droplets. Toluidine blue **(A,B,E,F,I,J,M,N)**, scale bar: 20 μm. Electron microscopy **(C,G,K,O)**, scale bar: 2 μm. Masson’s stain **(D,H,L,P)**. Scale bar: 20 μm.

**FIGURE 7:**
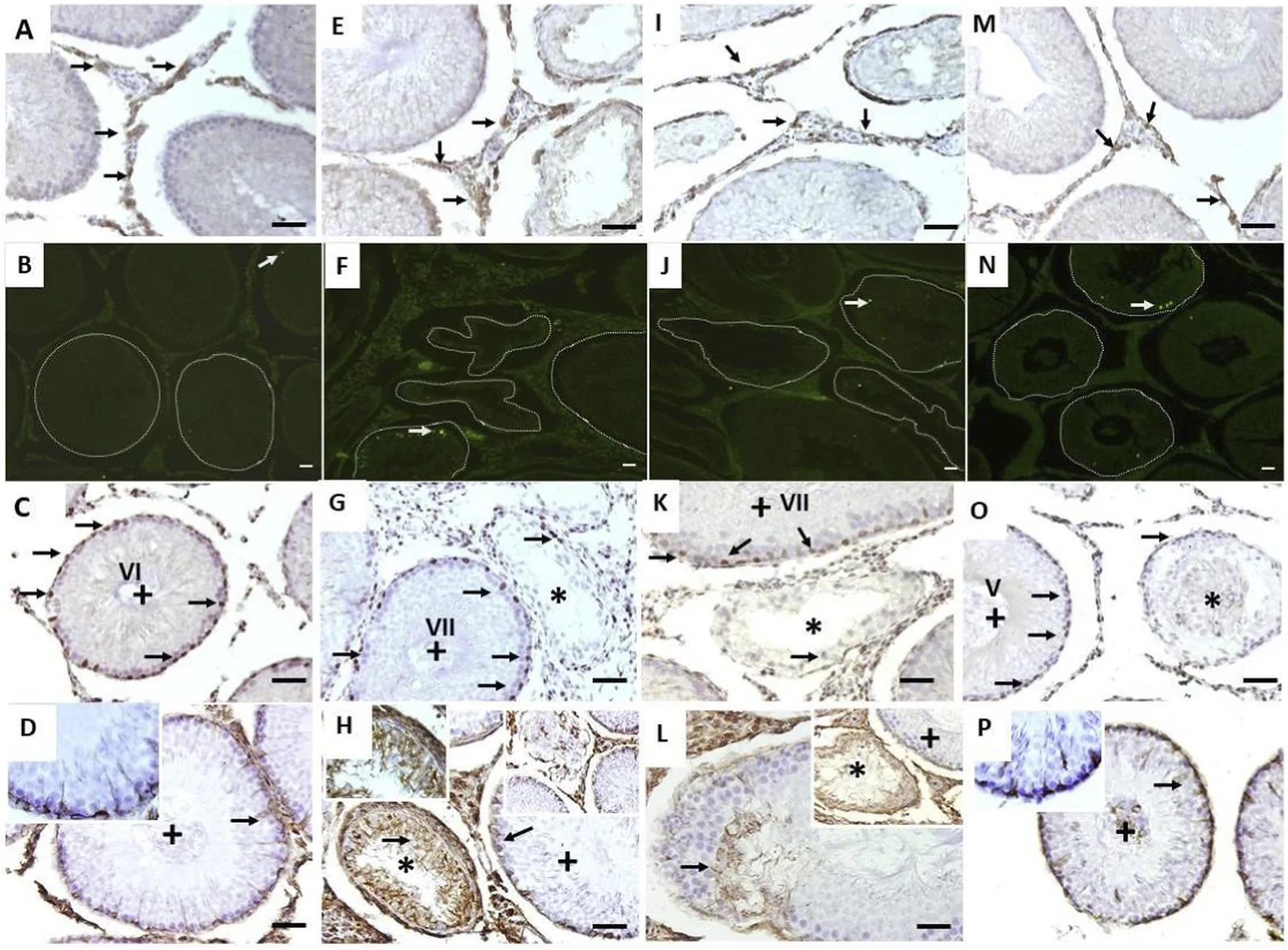
Seminiferous tubes from 190 dpp. Immunohistochemistry for the HSD17B3 enzyme: in the Control group **(A)**, Leydig cells immunoreactive to the enzyme are visible (arrow); in the experimental groups LeuEP **(E)**, LeuAP **(I)**, and LeuPP **(M)**, immunoreactivity for the enzyme is clear (arrow), compared to 90 dpp. TUNEL assay to identify apoptotic cells: in the Control group **(B)**, the presence of apoptotic cells is almost nil; whereas in the experimental groups LeuEP **(F)**, LeuAP **(J)**, and LeuPP **(N)**, apoptotic cells are still observed, although they are scarce. Immunohistochemistry for AR: in the Control group **(C)**, the presence of AR is clear; with recovery at 190 dpp, the LeuEP **(G)**, LeuAP **(K)**, and LeuPP **(O)** groups show greater immunoreactivity to AR in the recovered tubules, but not in the tubes that remained atrophied. Immunohistochemistry for vimentin: in the Control group **(D)**, immunoreactivity to vimentin was observed perpendicular to the basement membrane; in the experimental groups LeuEP **(H)** and LeuAP **(L)**, disorganization of vimentin immunoreactivity was observed in the atrophic tubes (*) in contrast to the tubes with spermatogenesis (+); in the LeuPP **(P)** group, vimentin organization was observed in most of the tubes. The stages of the seminiferous epithelium cycle are shown in roman numerals. Scale bar: 50 μm.

The Control group maintained complete spermatogenesis with healthy tubes, as described for 90 dpp (Figures 6A–D). The seminiferous tubes in the experimental groups exhibited a variety of patterns. In the LeuEP and LeuAP groups, tubes were observed in which atrophy persisted, accompanied by vacuolization, germ cell hypoplasia, and peritubular cell hyperplasia, as well as Sertoli cell nuclei with deep indentations and even SCO (Figures 6E–G,I-K). In the tubes that did not recover, the increased thickness of the tubular wall persisted; this wall was composed of layers of cells, myoid peritubular cells, fibroblasts, and Leydig cells (progenitor/immature due to the presence of lipid droplets), along with collagen fibers and blood vessels (Figures 6E–L), this being observed primarily in LeuEP. The hyperplasia of the two morphologically described populations of Leydig cells at 90 dpp was preserved, exhibiting fine and multiple cytoplasmic extensions (Figures 6F,G,J) and a greater immunoreactivity to the enzyme, HSD17B3 (Figures 7A,E,I). It should be noted that tubes with restored spermatogenesis were also observed, containing spermatozoa and an increase in tubular size (Figures 6E,F,I,J). The LeuPP group showed a higher proportion of tubes with complete spermatogenesis, with few atrophied tubules (Figures 6M–P) and immunoreactivity to the HSD17B3 enzyme similar to that of the Control (Figure 7M).

When comparing the number of mast cells in the LeuEP group (12.21 ± 3.01), LeuAP group (11.60 ± 3.57) and LeuPP (1.95 ± 1.87) at 190 dpp with their 90 dpp counterparts, a reduction (p < 0.05) were observed in the LeuEP and LeuAP groups (Figure 3A; 4E, F), but not in the LeuPP groups, which maintained an increased number of mast cells. All experimental groups at 190 dpp, with the exception of LeuPP, showed a higher number of mast cells compared to the Control group (0.20 ± 0.20) (Figure 3A).

On the other hand, when analyzing the number of Leydig cells in the LeuEP (35.95 ± 6.86), LeuAP (41.70 ± 7.19), and LeuPP (23.85 ± 2.70) groups at 190 dpp compared to the 90 dpp time point, a reduction (p < 0.05) was observed in the LeuEP group (Figure 4G), but not in the LeuAP and LeuPP groups, which maintained a high number of Leydig cells. Furthermore, at 190 dpp, all experimental groups, with the exception of the LeuPP group, showed a higher number of Leydig cells compared to the control group (17.20 ± 1.90, Figure 3B).

#### 3.2.3 Apoptosis and proteins regulating Sertoli cell function

All experimental groups showed a decrease in the %Apoptosis at 190 dpp compared to the data obtained at 90 dpp; the LeuEP group decreased by 65%, the LeuAP group by 71%, and the LeuPP group by 65% (Figures 5D–F, p < 0.05). However, even after GnRHa was withdrawn, the LeuEP group did not reach the Control group’s values at 190 dpp (Table 2, p < 0.05; Figures 7B,F,J,N). The ARICN per tube in the Control group was 46.13 ± 1.59; all experimental groups maintained the same %ARICN reduction as at 90 dpp, without reaching the Control group’s value (Table 2, p < 0.05). The ARICN average per tube was 21.91 ± 2.71 for LeuEP, 24.28 ± 11.28 for LeuAP, and 29.04 ± 9.38 for LeuPP (Figures 7C,G,K,O).

In the Control group, vimentin was distributed similarly to that observed at 90 dpp. In the LeuEP and LeuAP groups, this distribution mirrored that of the Control in tubes with complete spermatogenesis, whereas it appeared disorganized in atrophic tubes. Even some tubes with complete spermatogenesis showed areas of vimentin disorganization (Figures 7D,H,L). The LeuPP group retained the distribution pattern of the Control group, showing only a few areas with loss of this distribution (Figure 7P).

## 4 Discussion

GD can cause anxiety and depression during the period of secondary sexual characteristic development; therefore, seeking treatments to delay puberty is considered supportive. It is noted that not all adolescents who decide to undergo puberty delaying treatment continue with this approach and eventually discontinue it (Giovanardi, 2017; Brik et al., 2020).

Leuprolide acetate is a GnRHa that has been used to delay puberty. Its chronic administration acts by inhibiting the HPT axis, which affects testicular hormone production and the molecules necessary for spermatogenesis (Oduwole et al., 2021). This can lead to dysfunction of Sertoli, Leydig, and myoid peritubular cells in the testis.

In this study, it was observed that the administration of leuprolide acetate during puberty, evaluated at 90 dpp, halted spermatogenesis with clear signs of degeneration. The severity of the effects depended on the timing of treatment initiation at 25 and 35 dpp (equivalent to Tanner stages II-III and IV-V) and 60 dpp (postpubertal). Withdrawal of leuprolide acetate for two spermatogenic cycles in the LeuEP and LeuAP groups partially reduced the testicular alterations (50%-60% healthy tubes). Spermatogenesis was nearly restored when the analog was administered postpubertally (85%-90% healthy tubes).

There are no reports evaluating testicular function in individuals with GD who have undergone puberty blockade. The closest study is a cohort study of 214 individuals assigned male at birth treated with triptorelin (starting in adolescence) or cyproterone acetate (in adulthood) combined with estrogens (initiated at age 16 or after age 18) and orchiectomy at age 18 or older, following at least 1 year of estrogen treatment. Histological and immunohistochemical analyses were performed on testicular tissue obtained during orchiectomy. Complete spermatogenesis was observed in 10 trans women (4.7%) who had initiated puberty blockade at Tanner stage IV or later. In contrast, individuals who began puberty blockade treatment at Tanner stage II or III showed immature germ cells (60%-70% spermatogonia), indicating greater damage in those who halted puberty at an earlier stage (de Nie et al., 2022).

Studies in rats administered leuprolide acetate at a dose of 25 μg/kg/day from 25 to 50 dpp show a slight reduction in testicular volume (Guarraci et al., 2021). However, these studies administered the agonist over a short period that did not span a complete spermatogenesis cycle. In similar studies conducted on adult animals, blockade of the HPT axis, reporting a decrease in hormone levels (FSH, LH and testosterone), testicular weight or volume, associated with a reduction in germ cell numbers. This decrease in testosterone production has been linked to the interruption of spermatogenesis (Oduwole et al., 2018), and an increase in apoptosis in leptotene spermatocytes and round spermatids in the elongation phase of spermiogenesis, so that spermatogenesis does not proceed beyond the round spermatid stage, this is reflected in an absence of spermatozoa, and some tubules lacking germ cells and therefore decline in sperm quality parameters associated with infertility (Bartlett et al., 1986; Pinski et al., 1993; O’Donnell et al., 1994; O’Donnell et al., 1996; Okada et al., 1994; Aslam et al., 1999; Roth et al., 2000; Hild et al., 2001; Bakalska et al., 2004; Zheng and Fulu, 2006; Wang et al., 2009; Eski et al., 2019; Marcos et al., 2025). It should be mentioned that FSH has been reported to be involved in the early stages of spermatogenesis, whereas testosterone is in later stages, when spermatocyte development begins (Oduwole et al., 2018; Wang et al., 2022), therefore, blocking the axis HPT, could have deleterious effects on the entire spermatogenic cycle. These reports are consistent with was observed in the LeuEP and LeuAP groups, although blocking the axis at puberty also caused alterations in the interstitial cells.

During early puberty (Tanner stages I and II), spermatogenesis is not yet complete. At this stage, important processes of differentiation and maturation are taking place, coordinated by Sertoli, Leydig, and myoid peritubular cells; therefore, blockade of the HPT axis may have different effects than when the blockade occurs in adulthood.

When the analog was administered during early puberty (LeuEP), the marked atrophy and tubular damage with spermatogenesis arrested at the spermatocyte stage, along with a reduced number of tubes containing AR-immunoreactive cells and low intensity of immunoreactivity to the HSD17B3 enzyme in Leydig cells, as observed in this study, indicated the absence of androgenic action. At this age, 25 dpp, progenitor and immature adult Leydig cells were still present (Supplementary Fig.), as described by Li et al. (2022). It is known that progenitor Leydig cells are mitotically active and gradually lose this capacity as they differentiate into more advanced stages (Li et al., 2022), with the presence of LH being critical for the development of adult Leydig cells (Ye et al., 2017). The absence of androgenic action due to GnRHa administration led to continued proliferation of this cell type, resulting in hyperplasia of progenitor Leydig cells that were arranged around the seminiferous tubes between collagen fibers, along with a thick basement membrane containing abnormal myoid peritubular cells. This created a mechanical barrier against the action of molecules external to the seminiferous tube. The presence of large lipid droplets in some of the peritubular cells, as observed under an electron microscope, suggests that these are progenitor Leydig cells (Wang et al., 2026). It has been demonstrated that in mice knockout for the AR- (ARKO), their peritubular cells proliferate uncontrollably due to the absence of androgen action (Tan et al., 2005) and there is a 60% reduction in adult Leydig cells (Ye et al., 2017). Therefore, proper signaling in peritubular myoid cells in response to androgens is important for regulating the development, structure, and normal function of mature adult Leydig cells.

The adult Leydig cells that managed to position themselves in the central part of the testicular interstitium that we observed, presented two morphologically different populations and one of them with long and thin cytoplasmic extensions, possibly compensating for the deficiency of the action of androgens. This type of morphological abnormality has been described in hemicastrated rats during puberty (Furuya, 1990).

The presence of two populations of Leydig cells has also been described by Welsh et al. (2012). They observed that the composition of lipid droplets differs between these populations, suggesting two distinct functional states in mice in which AR was selectively removed from myoid peritubular cells.

It is also known that, in humans, the Leydig and peritubular myoid cell lineages differentiate during the prepubertal/peripubertal stages (Guo et al., 2020). In addition, mice with AR knockout in peritubular myoid cells (PTM-ARKO) also exhibit alterations in the basement membrane, as observed in this study. This thickened basement membrane may have contributed to impeding the free passage of molecules from outside the seminiferous epithelium that are necessary for maintaining spermatogenesis (Russell et al., 1989).

Mast cell hyperplasia also contributed to the deposition of collagen fibers that intercalated among the peritubular cells, contributing to the formation of this physical barrier. Through the secretion of tryptase, chymase, histamine, TGF-β1, IL-13, IL-9, CCL2, PDGF, glycosaminoglycan, and FGF-2, mast cells participate in collagen fiber synthesis by stimulating fibroblasts (Zhang and Kurashima, 2021; Himelreich-Perić et al., 2022). It has also been documented that an increase in the number of testicular mast cells in humans leads to disruption of spermatogenesis (Schmid et al., 2018), testicular histological abnormalities, SCO, fibrosis, a reduced seminiferous epithelium maturation index, and decreased vimentin expression (Hussein et al., 2005; Abdel-Hanid et al., 2018), leading to azoospermia and infertility (Apa et al., 2002).

Sertoli cells complete their maturation, androgen-dependent, at ~ 25 dpp in rats, corresponding to Tanner stages II-III in humans (Meroni et al., 2019; Guo et al., 2020; Rey, 2021); at this age, the first spermatocytes in the pachytene-diplotene stages were already present (Supplementary Fig.). In this regard, some authors have reported that FSH (in conjunction with testosterone) positively regulates the spermatogonial pool and their differentiation into spermatocytes, promoting entry into meiosis (Meroni et al., 2019). Therefore, inhibiting androgenic action with GnRHa not only resulted in thick tubular walls but also led to low AR immunoreactivity. Reduced or absent AR immunoreactivity has been reported when androgenic action is blocked in murine models (Turner et al., 2001; Hill et al., 2004). Furthermore, using testicular explants, it has been confirmed that AR expression in the physiological testicular microenvironment is directly upregulated by testosterone (Kamińska et al., 2024). In this way, Zhu et al. (2000) demonstrated that testosterone and LH suppression led to a gradual decrease in Sertoli cells AR expression throughout the administration of GnRHa treatment, becoming undetectable at 8 weeks. This led to Sertoli cell dysfunction, as demonstrated by the disorganization of vimentin immunoreactivity necessary for the formation of the branched scaffold. This disrupted spermatogenesis, causing marked atrophy and tubular damage with a large number of cells undergoing apoptosis. Additionally, in models lacking FSH or its receptor, the number of Sertoli cells decreases, affecting the number of germ cells in the seminiferous tubule, notably, these germ cells decreases even further at post-meiotic stages (Oduwole et al., 2018).

At 35 dpp, it has been reported that half of the adult Leydig cell population is immature and the rest is mature (Hardy et al., 1989; Li et al., 2022), consistent with the findings of this study at this age (Supplementary Fig.). Immature adult Leydig cells still possess proliferative capacity, which is gradually lost as they mature (Ge et al., 2005; Li et al., 2022). This proliferative capacity is halted by androgenic action (Tan et al., 2005). When puberty was inhibited at this age and continued until 90 dpp, there was an increase in the layers of cells and collagen fibers in the tubular wall, though to a lesser extent than when inhibition began at 25 dpp. The large number of mast cells present also promoted fibrosis, as mentioned earlier (Zhang and Kurashima, 2021; Himelreich-Perić et al., 2022).

At 35 dpp, the development of the asynchronous seminiferous tubes already included, in the most advanced first spermatogenic wave, spermatids in phase 13 of spermatogenesis (Supplementary Fig.). Impaired androgen action during puberty from 35 to 90 dpp was demonstrated by tubular atrophy and damage with increased apoptosis, arrest of spermatogenesis, increased tubular wall thickness, a reduced number of tubes with AR-immunoreactive cells, and low intensity of immunoreactivity to the HSD17B3 enzyme in Leydig cells, to a lesser extent than that reported for 25 dpp. This was likely due to the degree of differentiation of Leydig and germ cells at 35 dpp, with spermatids already present, indicating that the system was mature.

When leuprolide acetate was administered postpubertally (LeuPP) at 60 dpp and discontinued at 90 dpp, the layers of peritubular cells surrounding the seminiferous tubes were thinner compared to the groups that began blockade at 25 and 35 dpp (LeuEP and LeuAP). It has been documented in rats that by 56 dpp, the predominant population consists of mature adult Leydig cells, with few progenitor cells or immature adult cells that may still exhibit proliferative activity. By 60 dpp, testicular cell maturation had already been completed, and the first wave of spermatogenesis had already concluded in all seminiferous tubes (Supplementary Fig.). When leuprolide was administered for 30 days, testicular atrophy was less severe than when leuprolide administration began at 25 or 35 dpp (LeuEP and LeuAP).

Therefore, the responsiveness to the deficiency of androgen action on Sertoli, Leydig, and peritubular cells changes with the stage of development, differing between puberty and the postpubertal period.

By allowing a recovery period without the administration of leuprolide acetate, some parameters improved compared to the damage observed at 90 dpp in the LeuEP and LeuAP groups; however, they did not reach the levels seen in the Control group. In other words, the damage was permanent, particularly when puberty arrest was initiated at 25 dpp and 35 dpp, with 56.82% and 54.38%, respectively, of tubes with arrested spermatogenesis, in which SCO was observed, seminiferous tubes with thickened walls that possibly impeded the paracrine action of hormones and other regulatory molecules, vimentin disorganization, and low immunoreactivity to ARs despite an increase in immunoreactive intensity to HSD17B3. In these same experimental groups, seminiferous tubes showing clear recovery exhibited an increase in area, a higher degree of seminiferous epithelium maturation, and re organization in vimentin immunoreactivity, reducing epithelial damage, apoptosis, the number of mast cells, and tubular wall thickness, even though the deficiency in ARICN persisted. Leydig cell hyperplasia was preserved, to a lesser degree than at 90 dpp, with Leydig cells exhibiting fine cytoplasmic extensions. Leydig cells, which differed in shape, cell size, and lipid droplet size, may have exhibited different functional activity, as previously described (Welsh et al., 2012). Furthermore, the observed increase in HSD17B3 immunoreactivity may have resulted from the re-establishment of the HPT axis, wherein LH acted upon LHCGRs to drive Leydig cell maturation by triggering cAMP/PKA signaling (Wang et al., 2026). However, not all Leydig cells exhibited immunoreactivity to the HSD17B3 enzyme; this was possibly due to dysfunction in Sertoli and peritubular myoid cells, which are known to contribute local paracrine factors, such as growth factors (e.g., IGF-1, Desert Hedgehog), required for the maturation of immature adult Leydig cells (Wang et al., 2026).

Our data are consistent with what has been reported in adult models evaluating long-tem effects following the discontinuation of the GnRHa. Where a failure in the recovery of testicular size, sperm parameters, the irregular cellular arrangement within seminiferous tubules or the coexistence of normal and atrophied tubules (Lehrer et al., 1992; Hild et al., 2001; Eşki et al., 2019), despite the recovery in testosterone levels (Pinski et al., 1993; Eşki et al., 2019; Hild et al., 2001). It has also been reported that prolonged suppression of spermatogenesis in monkeys may be irreversible, unlike what has been observed in murine models (Boekelheide et al., 2005). Therefore, it is of great importance to evaluate the long-term effect of the use of analogues in the human pubertal population.

The reduction in apoptosis may be due to androgenic action, since numerous studies have confirmed that the action of testosterone via the AR in Sertoli cells determines the expression of genes governing the development and survival of germ cells (Cao et al., 2021).

On the other hand, LIF produced by myoid peritubular cells and glia-derived neurotrophic factor (GDNF), secreted by Sertoli and peritubular cells with the involvement of testosterone, promote the survival and proliferation of spermatogonial stem cells (SSC) (Piquet-Pellorce et al., 2000; Chen et al., 2016; Mayerhofer et al., 2018). It is possible that morphological abnormalities in myoid peritubular and Sertoli cells, as well as reduced androgen action, impeded the differentiation and survival of SSCs, resulting in seminiferous tubes with SCOs, as observed at 190 dpp.

Temporary administration of leuprolide acetate in postpubertal animals, which did not cause the same degree of damage as in the LeuEP and LeuAP groups, resulted in clear recovery, with only 20.25% of tubes showing arrested spermatogenesis. This was possibly due to the fact that, when administration of the analog began, spermatogenesis was already complete, with mature adult Leydig cells and fully functional Sertoli cells, which were less sensitive to the decrease in androgens.

## 5 Conclusion

Therefore, this is the first report demonstrating that temporary arrest of puberty initiated at 25 and 35 dpp (equivalent to Tanner stages II-III and Tanner stages IV-V, respectively) results in permanent testicular alterations, unlike in animals where GnRHa was administered postpubertally. Adolescents and their parents/guardians should be aware that there is no solid information regarding the safety of puberty blockers with respect to fertility in adulthood. This area should be explored in humans to inform clinical decision-making.

## Data Availability

The raw data supporting the conclusions of this article will be made available by the authors, without undue reservation.
