# Supplementary material for: Long-term testicular changes caused by the temporary suspension of puberty initiated at different stages of development
Source: Front Cell Dev Biol. 2026 Aug 31;14:1936689. doi: 10.3389/fcell.2026.1936689 (PMC13572694; doi:10.3389/fcell.2026.1936689)
Supplement: Supplementary file 1 [file DataSheet1.docx]

Supplementary Material

**Supplementary Figure 1.** Seminiferous tubes at 25 dpp, 35 dpp, and 60 dpp, showing the histological stage at which leuprolide acetate was administered in the LeuEP, LeuP, and LeuPP groups, respectively. At 25 dpp (A, B), the most advanced germ cells observed are spermatocytes in the pachytene/diplotene stages (Sc) and elongated cells surrounding the seminiferous tube, possibly progenitor Leydig cells (PLCs) in the process of migrating to the tubular interstitium. In the interstitium, immature adult Leydig cells (IALC) with their characteristic oval shape are observed. At 35 dpp (C, D), the most advanced germ cells are spermatids in stage 13 (Sd) of spermatogenesis. In the interstitium, mature adult Leydig cells (MALC) with their characteristic polyhedral shape and immature adult Leydig cells (IALC) are observed. At 60 dpp (E, F), complete spermatogenesis is observed. In the interstitium, mature adult Leydig cells (MALC) with their characteristic polyhedral shape are observed. Toluidine blue. Scale bar: 20 μm.

.
